# DYRK1A modulates fear memory formation via epigenetic modification

**DOI:** 10.1186/s13041-025-01216-8

**Published:** 2025-05-19

**Authors:** Dae-Si Kang, Ja Wook Koo

**Affiliations:** 1 Emotion, Cognition and Behavior Research Group, Korea Brain Research Institute, Daegu, 41062 Republic of Korea; 2 Department of Brain Sciences, Daegu Gyeongbuk Institute of Science and Technology, Daegu, 42988 Republic of Korea

**Keywords:** Contextual fear conditioning, Histone modification, DYRK1A

## Abstract

Fear memory formation is crucial for survival, with the hippocampus playing a central role. This study investigates the behavioral and molecular aspects of fear memory formation, focusing on Dual-specificity tyrosine phosphorylation-regulated kinase 1 A (DYRK1A), a protein known to be critical for cognitive functions. Our results demonstrate that DYRK1A expression in hippocampal CA1 pyramidal neurons is downregulated after contextual fear conditioning (CFC). We also observed a decrease in DYRK1A binding to the *Maoa* promoter, suggesting its involvement in transcriptional regulation during fear memory formation. In subsequent experiments, we modulated DYRK1A expression using viral vectors. DYRK1A overexpression reduced freezing behavior, while knockdown enhanced it. At the molecular level, DYRK1A overexpression resulted in elevated H3K4me3 levels, while knockdown decreased it. These findings indicate that DYRK1A regulates fear memory formation via epigenetic modifications, altering H3K4me3 levels and influencing *Maoa* transcription in the hippocampus. This research highlights the nuclear role of DYRK1A and suggests its potential as a therapeutic target for neuropsychiatric disorders related to fear and memory.

**Supplementary Information:**

The online version contains supplementary material available at 10.1186/s13041-025-01216-8.

## Introduction

In nature, fear memory represents one of the most highly conserved mechanisms across species, enabling animals to recognize and avoid environmental cues associated with potential threats or harmful conditions [1]. This essential adaptive function plays a critical role in survival and has been extensively studied, providing profound insights into the molecular and neural circuits involved in fear memory.

The hippocampus is a pivotal brain region involved in fear memory formation, consolidation, and retrieval [2]. It serves as an integrative hub that processes contextual information and interacts dynamically with other regions, such as the amygdala and prefrontal cortex to regulate cognitive and emotional responses. Emerging evidence suggests that hippocampal gene expression plays a crucial role in modulating its functional capacity, particularly in learning and memory.

DYRK1A has been implicated in various biological processes, including neuronal development and synaptic plasticity. In the hippocampus, DYRK1A plays a critical role in regulating neural activity and synaptic plasticity, influencing long-term potentiation and CFC [3]. Recent studies have linked DYRK1A to cognitive functions, suggesting its potential role in learning and memory. Notably, transcriptomic analysis (GSE214838) reveals that DYRK1A expression is downregulated in the hippocampus following fear conditioning, indicating a possible involvement in memory formation [4, 5].

Cognitive functions, including memory formation, are significantly influenced by epigenetic mechanisms, particularly histone modifications that regulate gene expression [6, 7]. DYRK1A is known to phosphorylate the C-terminal domain of RNA polymerase II, a critical step for transcriptional elongation, indicating its involvement in gene regulation. Given that histone methylation, particularly H3K4me3, serves as a key epigenetic marker linked to transcriptional elongation and memory formation, DYRK1A may modulate chromatin dynamics to influence transcription [8, 9].

We found that DYRK1A regulates transcription of genes involved in neurotransmitter metabolism, supporting this hypothesis. Specifically, our study reveals that DYRK1A binding to the monoamine oxidase A (MAOA) promoter, a key enzyme involved in the metabolism of monoamine neurotransmitters such as serotonin and norepinephrine, regulates gene expression critical for emotional learning and behavior. Given that *MAOA* deficiency leads to elevated monoamine levels and enhanced fear memory formation [10], DYRK1A may play a role in modulating synaptic plasticity through epigenetic mechanisms. Our results revealed a significant decrease in DYRK1A binding at the *MAOA* promoter following CFC, indicating that DYRK1A may regulate chromatin accessibility and influence *MAOA* expression, potentially impacting fear memory formation through modulation of neurotransmitter metabolism. However, the specific role of DYRK1A in epigenetic regulation during fear memory formation remains largely unexplored. This study aims to elucidate the role of DYRK1A in fear memory formation by examining its molecular functions in chromatin remodeling and gene transcription.

## Results

**Fig. 1:**
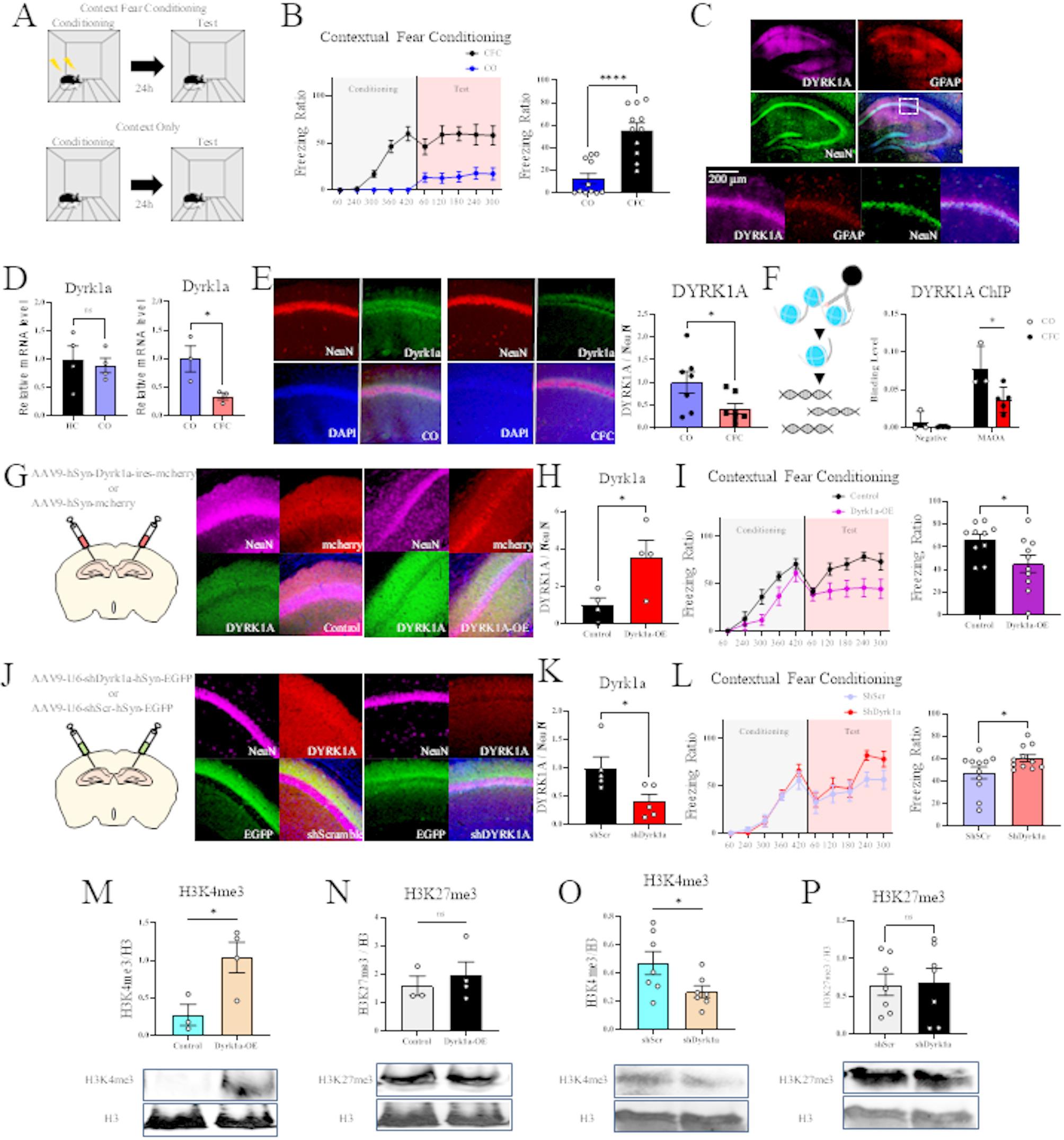
DYRK1A-mediated epigenetic regulation of fear memory formation A. Schematic of contextual fear conditioning B. Freezing behavior in CO and CFC groups. Data are presented as mean ± SEM (*n* = 11 per group; Student’s *t*-test; *P* < 0.0001) C. Representative images showing DYRK1A expression in the CA1 region. DYRK1A (purple), GFAP (red), NeuN (green); scale bar, 200 μm D. Quantitative PCR analysis of *Dyrk1a* mRNA expression in the hippocampus. Left: comparison between HC and CO groups (*n* = 4 per group; Student’s *t*-test; *P* = 0.7094). Right: comparison between CO and CFC groups (*n* = 3 per group; Student’s *t*-test; *P* = 0.0487). Data are shown as mean ± SEM E. Left: representative hippocampal images after CFC or CO. NeuN (red), DYRK1A (green), DAPI (blue). Right: quantification of DYRK1A levels (*n* = 7 per group; Student’s *t*-test; *P* = 0.0491) F. Left: schematic of ChIP-qPCR design. Right: DYRK1A binding at the *MAOA* promoter and negative control regions in CO and CFC groups. Data are shown as mean ± SEM (CFC, *n* = 3; CO, *n* = 5; *MAOA* promoter: Student’s *t*-test, *P* = 0.013303; negative region: Mann–Whitney *U* test, *U* = 5, *P* = 0.5714) G. Left: schematic of AAV-mediated DYRK1A overexpression in the hippocampus. Right: representative images of AAV-Syn-mCherry or AAV-Syn-DYRK1A-ires-mCherry injection H. Quantification of DYRK1A levels following overexpression (*n* = 4 per group; Student’s *t*-test; *P* = 0.039) I. Freezing behavior in DYRK1A overexpression and control groups (*n* = 10 per group; Student’s *t*-test; *P* = 0.0289) J. Left: schematic of AAV-mediated DYRK1A knockdown in the hippocampus. Right: representative images of AAV-U6-shScramble-Syn-EGFP or AAV-U6-shDYRK1A-Syn-EGFP injection K. Quantification of DYRK1A levels following knockdown (*n* = 5 per group; Student’s *t*-test; *P* = 0.0328) L. Freezing behavior in DYRK1A knockdown and scramble groups (*n* = 11 per group; Student’s *t*-test; *P* = 0.0414) M. H3K4me3 levels in the hippocampus of DYRK1A-overexpressing versus control mice (control, *n* = 3; overexpression, *n* = 4; Student’s *t*-test; *P* = 0.0366) N. H3K27me3 levels in the hippocampus of DYRK1A-overexpressing versus control mice (control, *n* = 3; overexpression, *n* = 4; Mann–Whitney *U* test, *U* = 5, *P* = 0.8571) O. H3K4me3 levels in the hippocampus of DYRK1A knockdown versus scramble groups (*n* = 7 per group; Student’s *t*-test; *P* = 0.0467) P. H3K27me3 levels in the hippocampus of DYRK1A knockdown versus scramble groups (*n* = 7 per group; Student’s *t*-test; *P* = 0.895)

To investigate the role of DYRK1A in fear memory formation, we conducted CFC (Fig. 1A). The CFC group showed significantly higher freezing rates compared to the context-only (CO) group (Fig. 1B), confirming successful fear memory formation. Histological analysis revealed that DYRK1A was primarily localized in pyramidal neurons within the CA1 region of the hippocampus (Fig. 1C). In contrast, GFAP-labeled astrocytes exhibited noticeably lower DYRK1A immunoreactivity, suggesting that DYRK1A expression is enriched in neurons rather than astrocytes.

Furthermore, to investigate DYRK1A regulation associated with fear memory, we performed qPCR analyses. First, we compared *Dyrk1a* mRNA expression between Home Cage (HC) and CO groups and found no significant difference, suggesting that context exposure alone does not affect *Dyrk1a* levels. In an additional analysis, we compared CO and CFC groups and observed a significant decrease in *Dyrk1a* mRNA expression (Fig. 1D), accompanied by a corresponding reduction in DYRK1A protein levels as assessed by histological analysis (Fig. 1E), indicating that fear memory formation is associated with downregulation of DYRK1A in the hippocampus.

ChIP-qPCR analysis revealed a significant reduction in DYRK1A binding at the *Maoa* promoter following CFC (Fig. 1F). This suggests that DYRK1A may influence chromatin accessibility and regulate *Maoa* transcription in response to fear conditioning.

To investigate the function of DYRK1A, we used viral vectors to modulate its expression in the hippocampus. We designed constructs for overexpression, such as AAV-hSyn-DYRK1A-ires-mCherry, and a control construct, AAV-hSyn-mCherry. For knockdown, we used AAV-U6-shDYRK1A-hSyn-EGFP, with AAV-U6-shScramble-hSyn-EGFP as the control. These constructs allowed us to investigate its role in behavioral regulation.

In the DYRK1A overexpression condition, we observed a significant increase in hippocampal DYRK1A levels, which was accompanied by a decrease in freezing behavior compared to the control group (Fig. 1G–I). Conversely, in the DYRK1A knockdown condition, hippocampal DYRK1A levels were reduced, and the freezing behavior was heightened, indicating enhanced fear memory retention (Fig. 1J–L). These findings strongly implicate DYRK1A as a key molecular regulator of fear memory.

Histone modification analysis showed that DYRK1A overexpression increased trimethylated histone 3 lys 4 (H3K4me3) levels, whereas knockdown led to a significant reduction (Fig. 1M–P). Interestingly, trimethylated histone 3 lys 27 (H3K27me3) levels remained unchanged, indicating that DYRK1A selectively modulates active histone markers rather than repressive ones. These results suggest that DYRK1A influences fear memory formation through epigenetic modifications, specifically by modulating histone balance and regulating the promoter activity of memory-related genes.

## Discussion

This study identifies DYRK1A as a key regulator of fear memory formation through transcriptional and epigenetic regulation. Our findings suggest that DYRK1A refines chromatin accessibility, potentially influencing *Maoa* expression and neurotransmitter metabolism, which may have implications for neuropsychiatric disorders related to fear and memory. Notably, we observed that DYRK1A inhibition reduces H3K4me3 levels in hippocampal neurons, which contrasts with recent findings in cancer cells, showing an increase in H3K4me3 levels upon DYRK1A inhibition [11]. This discrepancy likely reflects the context-dependent functions of DYRK1A in epigenetic regulation across different cell types and biological processes. While fear conditioning itself increases H3K4me3 at memory-related promoters [12], our study shows that both knockdown and overexpression of DYRK1A impair fear memory, despite having opposite effects on H3K4me3 levels. This suggests that DYRK1A does not function as a global H3K4me3 regulator but instead fine-tunes epigenetic states at specific loci critical for memory formation. These findings suggest that the concentration of DYRK1A and the cellular context are key determinants of memory-associated transcriptional regulation. Future studies should dissect the gene-specific and temporal dynamics of DYRK1A-mediated chromatin modulation and functional interactions with other epigenetic regulators.

## Electronic supplementary material

Below is the link to the electronic supplementary material.

Supplementary Material 1

## Data Availability

No datasets were generated or analysed during the current study.

## Abbreviations

AAV: Adeno-associated virus
CFC: Contextual fear conditioning
CO: Contextual only
ChIP: Chromatin immunoprecipitation
qPCR: Quantitative real time polymerase chain reaction
DYRK1A: Dual-specificity tyrosine-phosphorylation-regulated kinase 1 A
MAOA: Monoamine oxidase A
