# Supplementary material for: DYRK1A modulates fear memory formation via epigenetic modification
Source: Mol Brain. 2025 May 19;18:45. doi: 10.1186/s13041-025-01216-8 (PMC12090515; doi:10.1186/s13041-025-01216-8)
Supplement: Supplementary file 1 — Supplementary Material 1 [file 13041_2025_1216_MOESM1_ESM.docx]

**Additional file**

DYRK1A regulates fear memory formation via epigenetic modulation in hippocampal neurons

Dae-Si Kang^1,2^, Ja Wook Koo^1,2*^

^1^Emotion, Cognition and Behavior Research Group, Korea Brain Research Institute, Daegu 41062, Republic of Korea

^2^Department of Brain Sciences, Daegu Gyeongbuk Institute of Science and Technology, Daegu 42988, Republic of Korea

^*^Correspondence to:

Ja Wook Koo, Ph.D. (jawook.koo@kbri.re.kr)

**This PDF file includes:**

Supplementary Materials and Methods

Supplementary Figures and Tables: Table S1

**Supplementary Materials and Methods**

**Mice**

All experimental procedures were conducted in compliance with guidelines set by the Institutional Animal Care and Use Committee of the Korea Brain Research Institute.Adult male C57BL/6N mice, aged 8–10 weeks, were used in this study. Animals were housed in the Laboratory Animal Center under controlled conditions: 12-hour light/dark cycle (lights on at 08:00 am), temperature maintained at 22 ± 2 °C, and humidity at 50 ± 10%. Mice had unrestricted access to food and water throughout the study.

**Contextual fear conditioning**

Behavioral tests were conducted using a fear conditioning system (Panlab Harvard Apparatus). The test chamber (250 × 250 × 250 mm) was housed within a sound-attenuating box (670 × 530 × 550 mm). The fear conditioning protocol utilized a context with black walls and a metallic grid floor. On the conditioning day, mice were first allowed to acclimate to the context for 3 minutes. Subsequently, they received four aversive foot shocks (0.8 mA, 2 s duration) with 1-minute inter-trial intervals. The following day, test sessions were conducted in the same context, without electric foot shocks. During these sessions, mice were exposed to the context for 5 minutes, and their behavior was recorded for analysis. Freezing behavior was measured to evaluate fear reinstatement in the fear chamber (Fig. 1A-B).

**Chromatin immunoprecipitation (ChIP)**

Mice were sacrificed 2 hours after the fear memory test, and their brains were extracted and treated with cross-linking buffer [0.1 M NaCl, 1 mM EDTA, 0.5 mM EGTA, and 25 mM Hepes-KOH (pH 8.0)] containing 1% formaldehyde to fix the cells for 10 min at room temperature. For quenching of cross-linking, glycine (final concentration 125 mM) was used for 5 min at room temperature, and cells were harvested in ice-cold phosphate-buffered saline containing the protease inhibitor. Corrected tissues were lysed in ice-cold lysis buffer [140 mM NaCl, 1 mM EDTA (pH 8.0), 50 mM Hepes-KOH (pH 7.5), 10% glycerol, and 0.6% IGEPAL CA630, and protease inhibitors]. Nuclei extraction was performed by sonication with SDS lysis buffer [10% SDS, 1M Tris-HCl (pH 8.1), and 0.5 M EDTA], followed by centrifugation at 13,200 RPM for 15 min at 4℃. The supernatant containing transcription factor binding DNA was incubated with anti-Dyrk1a (ab65220) overnight at 4℃. After incubation, immune complexes were isolated by agarose beads and washed twice with each of the row salt buffer [0.1% SDS, 1% Triton X-100, 2 mM EDTA, 20 mM Tris-HCl (pH 8.1), and 150 mM NaCl], high salt buffer [0.1% SDS, 1% Triton X-100, 2 mM EDTA, 20 mM Tris-HCl (pH 8.1), and 500 mM NaCl], and LiCl buffer [250 mM LiCl, 1% IGEPAL CA630, 1% sodium deoxycholate, 1 mM EDTA, and 10 mM Tris (pH 8.1)]. The washed immune complexes were eluted from the agarose beads, and reverse cross-linking was conducted at 65℃ overnight. The DNA-containing eluate was treated with RNase A for a single hour at 37℃ and proteinase K for another couple of hours at 37℃. The DNA was purified by phenol: chloroform: isoamyl alcohol, followed by a PCR purification kit (Qiagen, Hilden, Germany).

**Stereotaxic surgeries**

Stereotaxic surgeries were performed as previously described. Mice were anesthetized with an intraperitoneal injection of ketamine (100 mg/kg) and xylazine (10 mg/kg) in 0.1M PBS. Anesthetized mice were secured in a stereotaxic frame. Injections were made using a 5-μL Hamilton microsyringe (7641-01) with a 33-gauge needle (7762-06). Viral vectors were injected at 200 nL/min, and the needle was left in place for 3 minutes post-injection before withdrawal. The incision was closed with surgical suture and tissue adhesive (Vetbond, 3M). All coordinates were relative to bregma. Mice recovered for at least two weeks before further procedures or behavioral testing.For DYRK1A overexpression (Fig. 1F-H), C57BL/6N mice received bilateral 1 μL injections of AAV9-Syn-Dyrk1a-ires-mcherry or AAV9-hSyn-mcherry (control) into the hippocampus (AP: -1.7 mm, ML: ±1.2 mm, DV: -1.5 mm, 10º angle).For DYRK1A knockdown (Fig. 1I-K), C57BL/6N mice received bilateral 1 µL injections of AAV9-U6-shDyrk1a-hSyn-EGFP or AAV9-U6-shScr-hSyn-EGFP (control) into the hippocampus using the same coordinates.

**AAV production and purification**

We utilized AAV9-Syn-Dyrk1a-ires-mcherry, AAV9-hSyn-mcherry, AAV9-U6-shDyrk1a-hSyn-EGFP, and AAV9-U6-shScr-hSyn-EGFP. Serotype 9 AAVs were produced and purified using a triple plasmid co-transfection protocol. AAV plasmids were transfected along with AAV-RepCap and pHelper plasmids into the AAVpro® 293T Cell Line (Takara, 632273). Three days post-transfection, cells were harvested by centrifugation at 1,000 RPM for 5 minutes. Cell pellets underwent three freeze-thaw cycles in AAV lysis buffer (100 mM Tris-HCl pH8.0, 150 mM NaCl). Following lysis, samples were treated with benzonase nuclease (Merck, E1014-5KU) at 37°C for 30 minutes and centrifuged at 4,000 RPM for 20 minutes.The AAV-containing supernatant was further purified by Opti-Prep (iodixanol; Merck, D1556-250ML) step gradient ultracentrifugation in a Beckman Type70Ti rotor at 50,000 RPM for 2 hours. The 40% gradient fraction containing AAV was collected and buffer-exchanged to PBS using Amicon Ultra-4 centrifugal filters (Millipore, UFC803024). Viral titers were determined by qPCR using primers targeting the fluorescent protein coding sequence: syn forward primer, 5'-tgcctacctgacgaccga-3'; syn reverse primer, 5'-ctctctgataggggatgcgc-3'.

**Histology**

Two hours after the fear memory test, mice were euthanized and perfused with 4% paraformaldehyde (PFA). Brains were removed, post-fixed in 4% PFA overnight, then cryoprotected in 30% sucrose at 4°C. Using a Leica cryostat, 50 µm thick sections were cut. Sections were blocked for 1 hour in PBS containing 2% normal donkey serum (Jackson ImmunoResearch, 017-000-121) and 0.2% Tween-20. Primary antibodies (anti-DYRK1A, Abcam AB65220; anti-NeuN, Merck MAB377) were diluted in blocking solution and incubated with sections overnight at 4°C. After three 10-minute PBS washes, sections were incubated for 3 hours with fluorescent secondary antibodies (anti-rabbit Alexa Fluor 488, Invitrogen A-21206; anti-rabbit Alexa Fluor 555, Invitrogen A-31570; anti-mouse Alexa Fluor 647, Invitrogen A31571) in blocking solution. Sections were washed three times in PBS for 10 minutes each, then mounted using VectaMount permanent mounting medium or Vectashield with DAPI (Vector Laboratories). Images were captured using a Leica TCS SP8 confocal microscope. Following confirmation of colocalization with NeuN-positive DYRK1A, we quantified DYRK1A fluorescence intensity using ImageJ

**Western blot**

Two hours after the fear memory test, mice were euthanized and hippocampal tissues from mice were homogenized in RIPA buffer (50mM Tris-HCl pH 7.5, 150mM NaCl, 0.1% Triton X-100, 0.1% SDS, 5mM EDTA, 1mM PMSF). Samples underwent sonication (11 cycles, 30s on/30s off) at 4°C using a Diagenode sonicator (B01060010). Lysates were then centrifuged (12,700 RPM, 20 min, 4°C) and protein concentrations determined via BCA assay. Proteins were denatured in sample buffer (60mM Tris-HCl pH 6.8, 2% SDS, 25% glycerol, 5% β-mercaptoethanol, 0.1% bromophenol blue) at 95°C for 30 minutes. SDS-PAGE was used to separate proteins, which were then transferred to nitrocellulose membranes (BIO-RAD, 1620177). Membranes were blocked with 3% BSA in TBS-T for 1 hour at room temperature. Primary antibodies (anti-H3K27me3, Merck ABE44; anti-H3K4me3, diagenode C15410003; anti-H3, Cell signaling D2B12) were diluted in blocking solution and incubated with membranes overnight at 4°C or for 3 hours at room temperature. After washing with TBS-T, membranes were incubated with HRP-conjugated secondary antibodies in 5% skim milk/TBS-T for 1 hour at room temperature. Protein bands were visualized using an ECL chemiluminescence system and imaged with a Fujifilm LAS 4000 CCD camera. Band intensities were quantified using ImageJ software

**RNA extraction and quantitative real-time PCR Analysis**

Hippocampal tissues were harvested 1 hour after the fear memory test. Mice were euthanized and bilateral hippocampi were rapidly dissected on ice. All procedures were performed according to the manufacturer's guidelines. RNA concentration and purity were assessed spectrophotometrically using a NanoDrop 2000 (Thermo Fisher Scientific). cDNA was synthesized from purified RNA using the iScript™ cDNA Synthesis Kit (BioRad, 1708891). Reverse transcription reactions were carried out with random hexamer primers for cDNA synthesis. Quantitative PCR amplification was performed using the following primer pairs: GAPDH: Forward 5’-CATCACTGCCACCCAGAAGACTG-3’, Reverse 5’-ATGCCAGTGAGCTTCCCGTTCAG-3’; Dyrk1a: Forward 5’-GACCAAAGATGGAAAACGGGA-3’, Reverse 5’-CCTCCTGTTTCCACTCCAAGAA-3’.

**Statistical analysis**

The study incorporated multiple biological replicates. Animals were randomly allocated to injection and conditioned groups. Statistical analyses were performed using GraphPad Prism 10. Sample sizes (N) represent the number of animals used and are specified in the respective figure legends. Data are presented as mean ± standard error of the mean (SEM) or as individual data points. Detailed information regarding statistical methods, including specific tests used, p-values, and effect sizes, can be found in the figure legends and Supplementary Table S1.

**Supplementary Tables**

**Table S1.** Summary of statistical analyses.
